# Dysregulation of MicroRNAs and PIWI-Interacting RNAs in a *Caenorhabditis elegans* Parkinson’s Disease Model Overexpressing Human α-Synuclein and Influence of *tdp-1*

**DOI:** 10.3389/fnins.2021.600462

**Published:** 2021-03-08

**Authors:** Linjing Shen, Changliang Wang, Liang Chen, Garry Wong

**Affiliations:** ^1^Centre for Reproduction, Development and Aging, Faculty of Health Sciences, University of Macau, Macau, China; ^2^Bioland Laboratory (Guangzhou Regenerative Medicine and Health Guangdong Laboratory), Guangzhou, China; ^3^Department of Computer Science, College of Engineering, Shantou University, Shantou, China; ^4^Key Laboratory of Intelligent Manufacturing Technology of Ministry of Education, Shantou University, Shantou, China

**Keywords:** microRNA, PIWI-interacting RNA, neurodegenerative disease, synucleinopathies, *Caenorhabditis elegans*

## Abstract

MicroRNAs (miRNAs) and PIWI-interacting RNAs (piRNAs) regulate gene expression and biological processes through specific genetic and epigenetic mechanisms. Recent studies have described a dysregulation of small non-coding RNAs in Parkinson’s disease (PD) tissues but have been limited in scope. Here, we extend these studies by comparing the dysregulation of both miRNAs and piRNAs from transgenic *Caenorhabditis elegans* (*C. elegans*) nematodes overexpressing pan-neuronally human α-synuclein wild-type (WT) (HASN^WT^ OX) or mutant (HASN^A53T^ OX). We observed 32 miRNAs and 112 piRNAs dysregulated in HASN^A53T^ OX compared with WT. Genetic crosses of HASN^A53T^ OX PD animal models with *tdp-1* null mutants, the *C. elegans* ortholog of TDP-43, an RNA-binding protein aggregated in frontal temporal lobar degeneration, improved their behavioral deficits and changed the number of dysregulated miRNAs to 11 and piRNAs to none. Neuronal function-related genes *T28F4.5*, *C34F6.1*, *C05C10.3*, *camt-1*, and *F54D10.3* were predicted to be targeted by cel-miR-1018, cel-miR-355-5p (*C34F6.1* and *C05C10.3*), cel-miR-800-3p, and 21ur-1581 accordingly. This study provides a molecular landscape of small non-coding RNA dysregulation in an animal model that provides insight into the epigenetic changes, molecular processes, and interactions that occur during PD-associated neurodegenerative disorders.

## Introduction

MicroRNAs (miRNAs) and PIWI-interacting RNAs (piRNAs) are well conserved small non-coding RNAs 21–23 or 24–31 nucleotides in length, respectively. MiRNAs can regulate gene expression at the transcriptional level by pairing to the 3′ UTR of target mRNAs and inducing cleavage or posttranscriptional gene silencing (Hutvagner and Zamore, 2002; Bartel, 2009). PiRNAs mainly function in the repression of transposons at transcriptional or posttranscriptional levels to safeguard the germline genome (Iwasaki et al., 2015; Ernst et al., 2017). There is increasing interest in the role that miRNAs play in neuronal homeostasis and pathogenesis of neurodegenerative diseases via dysregulation, targeting of key genes, or altering the epigenetic landscape (Tan et al., 2015; Quinlan et al., 2017). The identification of miRNAs dysregulated in neurodegenerative diseases and construction of related animal models may thus aid in delineation of molecular mechanisms underlying their pathologic processes (Juzwik et al., 2019). Moreover, exploitation of these molecular pathways via precision therapy strategies for neurodegenerative diseases is well underway (Junn and Mouradian, 2012; Pereira et al., 2017; Nuzziello et al., 2019). While piRNAs have a well-established role in the protection of the germline genome, they also function in somatic cells including neurons (Lee et al., 2011; Yan et al., 2011). PiRNAs can participate in synaptic plasticity and axon regeneration of neurons (Rajasethupathy et al., 2012; Kim et al., 2018). Hundreds of piRNAs mainly originating from introns were found to be dysregulated in Alzheimer’s disease (AD) patient brains (Roy et al., 2017). In addition, piRNAs were also dysregulated in midbrain neuronal cells differentiated from skin fibroblasts of sporadic Parkinson’s disease (PD) patients similarly to PD postmortem brain samples (Schulze et al., 2018). Furthermore, pathogenic tau induced the reduction of piRNAs in tau-related neurodegenerative diseases such as AD and progressive supranuclear palsy and consequently caused abnormal transposable element activity and subsequently progressive neuronal death (Sun et al., 2018). PiRNAs are thus beginning to be appreciated as pathogenic factors and therapeutic targets for neurodegenerative diseases as well as miRNAs (Wakisaka and Imai, 2019).

α-Synuclein was initially identified as the predominant component in aggregates of Lewy bodies and Lewy neurites in PD patient neurons (Spillantini et al., 1997, 1998). Its mutation and overexpression were subsequently associated with familial PD (Polymeropoulos et al., 1997; Chartier-Harlin et al., 2004). Increasing evidence suggests that α-synuclein is also closely linked to other neurodegenerative diseases including dementia with Lewy bodies (DLB) and multiple system atrophy (MSA), which are termed synucleinopathies together with PD (Wakabayashi et al., 1998; Wong and Krainc, 2017; Visanji et al., 2019). Furthermore, α-synuclein aggregates are found in the abnormal deposition in amyotrophic lateral sclerosis (ALS) and AD frequently (Wilhelmsen et al., 2004; Korff et al., 2013; Takei et al., 2013). Pathologic α-synuclein could impair mitochondria and elevate the reactive oxygen species level, induce endoplasmic reticulum stress, inhibit proteasome activity, and cause cell death via activating caspase-3, caspase-9, and caspase-12 in PD (Smith et al., 2005; Wong and Krainc, 2017). Some studies showed that α-synuclein pre-fibrillar oligomers were likely the toxic species and that α-synuclein fibrils might be protective to neurons (Tanaka et al., 2004; Karpinar et al., 2009; Winner et al., 2011). Other studies showed that α-synuclein fibrils caused more dopaminergic neuron impairment or loss than oligomers due to their ability to disrupt cell membrane permeability (Peters et al., 2012; Taschenberger et al., 2012). Despite many years and a great deal of effort since its discovery, mechanisms underlying the toxicity of α-synuclein in neurodegenerative diseases continue to be developed and defined. As proteopathic intracellular and extracellular α-synuclein is a key protein in various synucleinopathies, its targeting remains a viable and attractive therapeutic strategy (Luk et al., 2012; Recasens et al., 2014; Karpowicz et al., 2019).

The disrupted metabolism of α-synuclein is implicated in its pathogenesis. α-Synuclein A53T mutant protein was more stable than other mutants or wild-type (WT) forms either *in vitro* or *in vivo* (Li et al., 2004). The enhanced stability of this α-synuclein A53T protein could then increase its own protein aggregate or fibril formation (Conway et al., 2000; Lee et al., 2002). Subsequently, lysosomal autophagy, which is responsible for WT α-synuclein degradation, was impaired when α-synuclein A53T mutant protein bound the lysosomal membrane receptor, suggesting an intracellular mechanism by which aggregates can begin accumulating (Cuervo et al., 2004). In our previous studies, human α-synuclein A53T overexpressing pan-neuronally (HASN^A53T^ OX) in *Caenorhabditis elegans* (*C. elegans*) could impair the locomotion, development, and basal slowing response; induce significant dopaminergic neuron degeneration; extend life span; and cause gene dysregulation. Human WT α-synuclein overexpressing pan-neuronally (HASN^WT^ OX) also impaired dopaminergic neurons, extended life span, and caused differential gene expression, but the locomotion, development, and basal slowing response were not affected to the same extent as HASN^A53T^ OX (Shen et al., 2020). Taken together, the toxicities of HASN^WT^ OX and HASN^A53T^ OX were different, while the underlying molecular process remains unexplored.

In this study, we hypothesized that miRNAs/piRNAs play important roles in the toxicities of HASN^WT^ OX and HASN^A53T^ OX in *C. elegans*. We previously observed that the deletion of *tdp-1*, the worm ortholog of human TDP-43 that is aggregated in frontal temporal lobar degeneration and ALS, could improve defects induced by HASN^WT/A53T^ OX in *C. elegans* (Shen et al., 2020). We further hypothesized that TDP-1 might support HASN^WT/A53T^ OX toxicity via sustaining the related miRNAs/piRNA expression. To test these hypotheses, we performed small RNA sequencing (sRNA-seq) on HASN^WT/A53T^ OX *C. elegans* and crosses with a *tdp-1* knock-out (*tdp-1* KO) strains. Here, we identified a large number of differentially expressed miRNAs and piRNAs (DE-miRNAs and DE-piRNAs). The predicted targets of these dysregulated small non-coding RNAs then indicate pathways that support the toxicity observed in these animal models.

## Materials and Methods

### *Caenorhabditis elegans* Strains

*C. elegans* strain N2 (WT) was obtained from the Caenorhabditis Genetics Center (CGC). Transgenic/mutant *C. elegans* strains constructed or crossed by our lab were as follows (Lakso et al., 2003; Shen et al., 2020): UM0010 (P*_*dat–1*_*::GFP, P*_*aex–3*_*::HASN^A53T^), human α-synuclein A53T overexpressing pan-neuronally (HASN^A53T^ OX); UM0011 (P*_*dat–1*_*::GFP, P*_*aex–3*_*::HASN^WT^), human α-synuclein WT overexpressing pan-neuronally (HASN^WT^ OX); UM0012 (P*_*dat–1*_*::GFP, *tdp-1* KO), *tdp-1* gene deletion strain; UM0013 (P*_*dat–1*_*::GFP, P*_*aex–3*_*::HASN^A53T^, *tdp-1* KO), generated from crossing of UM0010 and RB929 (*tdp-1* KO); and UM0014 (P*_*dat–1*_*::GFP, P*_*aex–3*_*::HASN^WT^, *tdp-1* KO), generated from crossing of UM0011 and RB929. The transgenic strains UM0010 and UM0011 had their transgenes integrated by X-ray irradiation and were back-crossed with WT N2 4× times (Vartiainen et al., 2006b).

### Small RNA Library Preparation and Sequencing

The *C. elegans* strains used for sRNA-seq were N2, UM0010, UM0011, UM0012, UM0013, and UM0014. Worms were collected at the L4 stage at 20°C. The total RNAs were isolated by TRIzol (Thermo Fisher Scientific, Waltham, MA, United States) after washing the worms three times with distilled water. Small RNA libraries were prepared according to the instructions of the NEBNext Multiplex Small RNA Library Prep Set for Illumina (New England Biolabs, Ipswich, MA, United States). The quantity and quality of the RNA and its transcribed cDNA were determined by an Agilent Bioanalyzer 2100 (Agilent Technologies, Santa Clara, CA, United States). The resulting cDNA libraries were then sequenced in triplicate on the Illumina HiSeq 2500 Platform in a single-end mode (50 nt).

### Identification of Differentially Expressed MicroRNAs and PIWI-Interacting RNAs and Novel MicroRNAs

The identification of DE-miRNAs and DE-piRNAs for comparisons was based on six strains assayed with the genotypes, as follows: WT, *tdp-1* KO, HASN^WT^ OX, HASN^A53T^ OX, HASN^WT^ OX + *tdp-1* KO, and HASN^A53T^ OX + *tdp-1* KO. The quality of reads was measured by fastQC, and the raw reads were trimmed using Trimmomatic (Bolger et al., 2014). For the miRNA analysis, trimmed reads were mapped to the genome using bowtie, and the genome sequence (WBcel235/ce11) was downloaded from the Ensembl database (Cunningham et al., 2019). The sam files were then converted to bam files and indexed using SAMtools (Li et al., 2009). The miRNA annotation file was downloaded from miRBase. FeatureCounts was used to measure the read count that mapped to a miRNA. “exactTest” from “edgeR” package was used to identify the DE-miRNAs with the threshold set as *p* value <0.05 and absolute fold change >2 (Robinson et al., 2010). The pipeline for identification of DE-piRNAs is similar to the identification of DE-miRNAs except that the genome data (WBcel235/ce11) and the piRNA annotation file are downloaded from the National Center for Biotechnology Information (NCBI) database^1^. Due to the large number of piRNAs, the threshold for the DE-piRNAs was set as *p* value <0.01, false discovery rate (FDR) <0.1, and absolute fold change >2.

The heatmaps for miRNAs/piRNAs expressed in different strains were drawn as described below. First, all expressed miRNAs/piRNAs were collected. The union set of DE-miRNAs (cutoff was *p* value <0.05 and absolute fold change >2) and DE-piRNAs (cutoff was *p* value <0.01, FDR <0.1, and absolute fold change >2) of mutant/transgenic strains compared with the WT strain was obtained. Then, the average read counts of all the miRNAs/piRNAs or the union set in different biological triplicates of each strain and the log2 transformed count-per-million (log2cpm) value were calculated. Finally, the *Z*-score of the log2cpm value of each miRNA/piRNA was calculated and used to draw the heatmap. The complete linkage method was used for hierarchical clustering analysis, which was performed by the “pheatmap” package. The novel miRNAs were identified with miRDeep2 (Friedlander et al., 2012).

### Enrichment Analysis of the Target Genes of Differentially Expressed MicroRNAs and Differentially Expressed PIWI-Interacting RNAs

The miRNA target information was downloaded from miRTarBase (Chou et al., 2018), and the piRNA target information was downloaded from piRTarBase (Wu et al., 2019). The Gene Ontology (GO) enrichment analysis in biological process (BP), molecular function (MF), and cellular component (CC) and Kyoto Encyclopedia of Genes and Genomes (KEGG) pathway enrichment analysis were performed using “enrichGO” and “enrichKEGG” functions, respectively, from the “clusterProfiler” package (Yu et al., 2012).

### The Construction of the Dysregulation Network

The information for the target genes of DE-miRNAs and DE-piRNAs was downloaded from the miRTarBase and piRTarBase using the stringent targeting rules, respectively. The data for transcription factors (TFs), regulating the transcription of miRNAs, were downloaded from the TransmiR database (Tong et al., 2019). The TFs and their regulated genes data were downloaded from the TF2DNA database (Pujato et al., 2014). The differentially expressed genes (DEGs) were obtained from RNA-Seq in our previous work with the criteria of *p* value <0.05 and absolute fold change >2 (Shen et al., 2020). Based on the interaction information, we constructed the dysregulation networks composed of DE-miRNA and DEG interactions, DE-piRNA and DEG interactions, and networks for TF and DE-miRNA interaction, for each comparison. The network construction was completed by the “igraph” package (Csardi and Nepusz, 2006).

### Identification of the Differentially Expressed MicroRNAs and Differentially Expressed PIWI-Interacting RNAs Targeting Neurodegenerative Disease-Associated Genes

In order to determine whether any DE-miRNA or DE-piRNA targets were associated with a neurodegenerative disease, we downloaded the curated gene–disease associations from the DisGeNET database (Pinero et al., 2020). The human genes associated with the neurodegenerative diseases including PD, AD, MSA, ALS, and Huntington’s disease (HD) were obtained from the DisGeNET database by using the keywords “Parkinson/Alzheimer/multiple system atrophy/amyotrophic lateral sclerosis/Huntington’s disease.” Then, the human homologue analysis for the target genes of the DE-miRNAs and DE-piRNAs in *C. elegans* was performed by the “homologene” package (Mancarci and French, 2019). Finally, the DE-miRNAs and DE-piRNAs targeting neurodegenerative disease-associated genes were identified by overlapping the homologue genes with the neurodegenerative disease associated genes.

### Quantitative Real-Time PCR

MiRNAs and piRNAs for qRT-PCR were selected from the DE-miRNAs (*p* < 0.05 and absolute fold change >2) and DE-piRNAs (*p* < 0.01, FDR < 0.1, and absolute fold change >2) lists generated from the sRNA-seq. The qRT-PCR for target genes was performed on the DEGs (*p* < 0.05 and absolute fold change >2) generated from our previous RNA-seq (Shen et al., 2020). Primer pairs for small RNAs had 3′ primers supplied by Mir-X miRNA qRT-PCR SYBR Kit (Takara Bio, Mountain View, CA, United States). The 5′ primers and the primer pairs for DEGs were designed and synthesized by BGI (Shenzhen, China) and are listed in Supplementary Table S1. The L4 age-synchronized N2, UM0010, UM0011, UM0012, UM0013, and UM0014 animals were collected (maintained at 20°C). Both total RNAs and small RNAs were isolated and purified by the mirVana^TM^ miRNA Isolation Kit (Life Technologies, Carlsbad, CA, United States). The target small RNAs were reverse transcribed and amplified by Mir-X miRNA qRT-PCR SYBR Kit (Takara Bio USA, Mountain View, CA, United States) and detected on the Applied Biosystems 7500 Fast Real-Time PCR System (Applied Biosystems, Foster City, CA, United States). Target genes were reverse transcribed with RevertAid Reverse Transcriptase (Thermo Fisher Scientific, Waltham, MA, United States), amplified using SYBR Green (Bio-Rad, Hercules, CA, United States), and detected on the Applied Biosystems 7500 Fast Real-Time PCR System (Applied Biosystems, Foster City, CA, United States). Three biological replicates in triplicate technical replicates were performed for each test. U6 and *act-1* were used as internal reference for target small RNAs and target genes, respectively, in *C. elegans*.

## Results

### HASN^A53T^ OX Strain Has More Dysregulated MicroRNAs/PIWI-Interacting RNAs Than Other Strains

**FIGURE 1:**
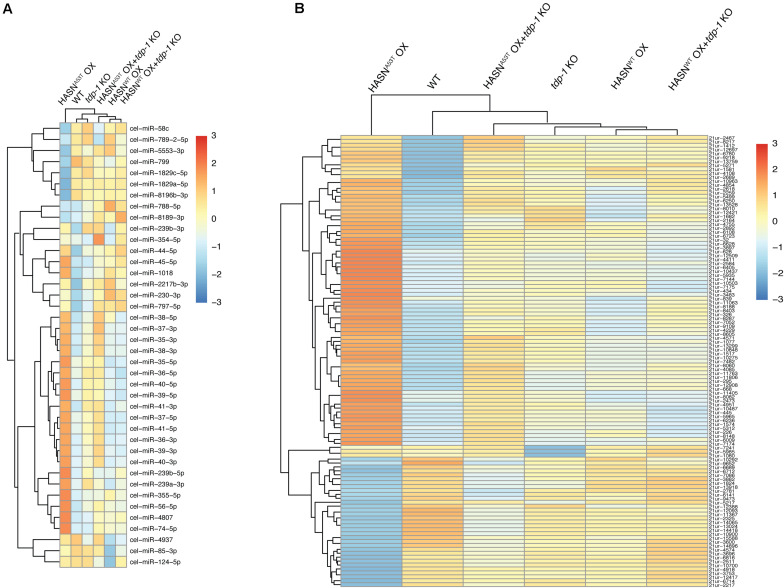
Hierarchical clustering for DE-miRNAs **(A)** and DE-piRNAs **(B)** of mutant/transgenic strains compared with WT strain. The cutoff of DE-miRNAs was *p* < 0.05 and absolute fold change >2; DE-piRNAs were *p* < 0.01, FDR < 0.1, and absolute fold change >2. The logarithmic scale represents the range of expression values of DE-miRNAs/DE-piRNAs. The top cluster dendrogram indicates the similarity of different strains based on the average expression values of all DE-miRNAs/DE-piRNAs of each strain. The left cluster dendrogram indicates the similarity of different DE-miRNAs/DE-piRNAs based on the average expression values of each DE-miRNA/DE-piRNA of all strains. The right colored bars show the DE-miRNA/DE-piRNA expression values.

A hierarchical clustering analysis of DE-miRNAs was performed to provide a global view on the differences between the strains (Figure 1A). The analysis showed three main groups: 1) HASN^A53T^ OX alone; 2) WT and *tdp-1* KO; and 3) HASN^WT^ OX, HASN^WT^ OX + *tdp-1* KO, and HASN^A53T^ OX + *tdp-1* KO. Hierarchical clustering of all the miRNAs showed a similar clustering profile of HASN^A53T^ OX alone, but differences with WT clustered with HASN^WT^ OX + *tdp-1* KO, HASN^A53T^ OX + *tdp-1* KO, and HASN^WT^ OX together with *tdp-1* KO (Supplementary Figure S1). A hierarchical clustering analysis of DE-piRNAs also showed HASN^A53T^ OX in its own cluster, with WT next, and then the rest of the strains together (Figure 1B). The read counts for miRNAs/piRNAs of different strains are listed in Supplementary Tables S2 and S3. The volcano plots of the miRNAs/piRNAs are shown in Supplementary Figures S2 and S3.

**FIGURE 2:**
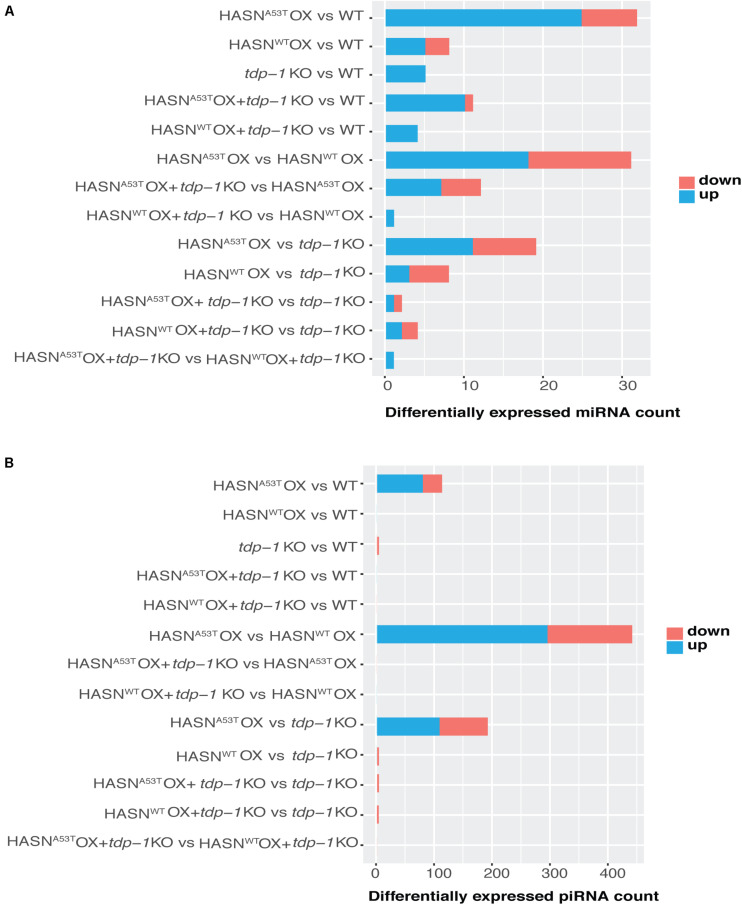
Counts of DE-miRNAs **(A)** and DE-piRNAs **(B)** generated from different comparisons. The cutoff of DE-miRNAs was *p* < 0.05 and absolute fold change > 2; that of DE-piRNAs was *p* < 0.01, FDR < 0.1, and absolute fold change > 2. The vertical lists show the comparisons. Horizontal bars indicate the DE-miRNAs/DE-piRNAs counts based on the bottom scales. Blue represents upregulated, and red represents downregulated.

In order to characterize the breadth and direction of differential expression between strains, we compared the DE-miRNAs (*p* < 0.05 and absolute fold change >2) and DE-piRNAs (*p* < 0.01, FDR < 0.1, and absolute fold change >2). Compared with WT, the expressions of miRNAs (Figure 2A) and piRNAs (Figure 2B) were changed significantly by HASN^A53T^ OX, which displayed 32 DE-miRNAs and 112 DE-piRNAs. Among these, over 2/3 of DE-miRNAs and DE-piRNAs were upregulated. The HASN^WT^ OX strain had far fewer, with eight DE-miRNAs and no DE-piRNAs. A total of 31 miRNAs and 440 piRNAs were differentially expressed when directly comparing HASN^A53T^ OX vs HASN^WT^ OX (Figures 2A,B).

### *tdp-1* Knock-Out Decreases the Number of Differentially Expressed MicroRNAs and Differentially Expressed PIWI-Interacting RNAs

We then analyzed the effect of the *tdp-1* KO. The loss of *tdp-1* caused a dramatic decrease in the number of DE-miRNAs and DE-piRNAs in HASN^A53T^ OX strain going from 32 to 11 and 112 to 0, respectively (Figures 2A,B). However, no such apparent difference was observed in HASN^WT^ OX + *tdp-1* KO strain compared with WT strain. HASN^A53T^ OX + *tdp-1* KO vs HASN^WT^ OX + *tdp-1* KO directly also resulted in fewer DE-miRNAs/DE-piRNAs going from 31 to 1 and 440 to 0, respectively (Figures 2A,B). These results demonstrate a clear effect of *tdp-1* KO to diminish the number of DE-miRNAs/DE-piRNAs between HASN^A53T^ OX and WT, and between HASN^A53T^ OX and HASN^WT^ OX. The DE-miRNAs and DE-piRNAs for the different comparisons are listed in Supplementary Tables S4 and S5. In addition, we also listed DE-piRNAs with *p* value < 0.01 and absolute fold change >2 in Supplementary Table S6 for reference.

### Gene Ontology and Kyoto Encyclopedia of Genes and Genomes Enrichment Analysis of Target Genes of Differentially Expressed MicroRNAs

**FIGURE 3:**
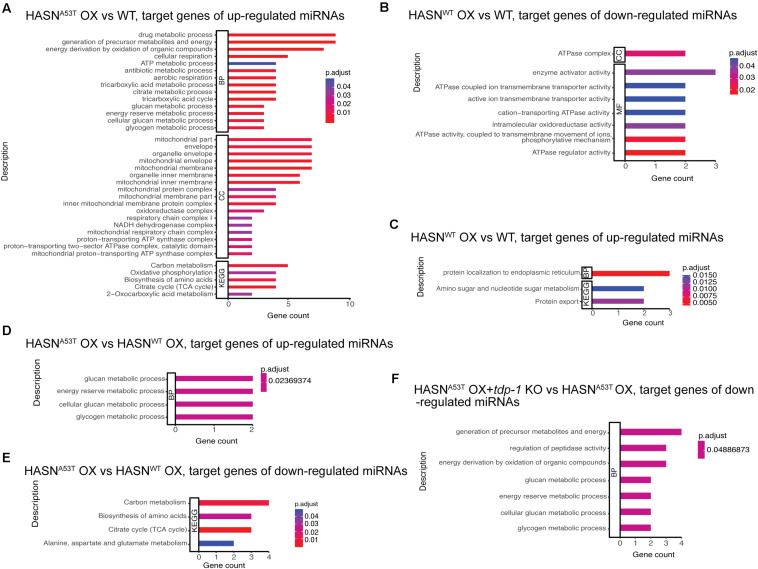
**(A–F)** GO and KEGG enrichment analysis for the target genes of DE-miRNAs from different comparisons. The cutoff of DE-miRNAs was *p* < 0.05 and absolute fold change >2. The bar length represents the counts of target genes corresponding to the vertical terms. The bar color represents the *p*-adjust value of each term. BP, biological process; CC, cellular component; MF, molecular function; KEGG, Kyoto Encyclopedia of Genes and Genomes.

To identify the functions of DE-miRNAs targets, we performed the GO and KEGG enrichment analysis from the comparisons: HASN^A53T^ OX vs WT, HASN^WT^ OX vs WT, HASN^A53T^ OX vs HASN^WT^ OX, *tdp-1* KO vs WT, HASN^A53T^ OX + *tdp-1* KO vs HASN^A53T^ OX, and HASN^WT^ OX + *tdp-1* KO vs HASN^WT^ OX. All the enrichment analysis results we obtained are shown in Figure 3. Upregulated miRNAs comparing HASN^A53T^ OX vs WT identified targets enriched for energy generation (carbon metabolism), biosynthesis of amino acids, drug metabolism, mitochondrial membrane, mitochondrial protein complex, and oxidative phosphorylation categories (Figure 3A). For down-/upregulated miRNAs comparing HASN^WT^ OX vs WT, the ATPase complex, ATPase coupled ion transmembrane transporter activity and intramolecular oxidoreductase activity, protein localization to endoplasmic reticulum, protein export, and amino sugar and nucleotide sugar metabolism were identified (Figures 3B,C). For comparisons of HASN^A53T^ OX vs HASN^WT^ OX, energy generation and amino acid metabolism were enriched (Figures 3D,E). Taken together, the functions of target genes of DE-miRNAs induced by HASN^A53T^ OX were different from those induced by HASN^WT^ OX, particularly in energy generation and amino acid metabolism. Furthermore, energy generation and biosynthesis of amino acids were enriched not only for the upregulated miRNAs comparing HASN^A53T^ OX vs WT but also for the downregulated miRNAs comparing HASN^A53T^ OX + *tdp-1* KO vs HASN^A53T^ OX (Figure 3F). This suggested that the expression of miRNAs associated with energy generation and amino acid metabolism might be supported by TDP-1 under the condition of HASN^A53T^ OX.

### Gene Ontology and Kyoto Encyclopedia of Genes and Genomes Enrichment Analysis of Target Genes of Differentially Expressed PIWI-Interacting RNAs

**FIGURE 4:**
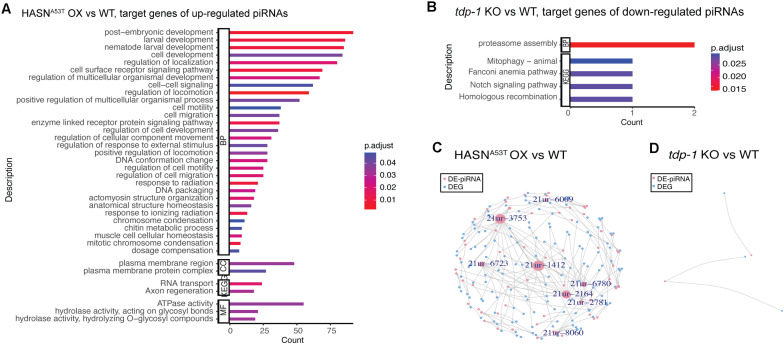
GO and KEGG enrichment analysis for the target genes of DE-piRNAs **(A,B)** and network of DE-piRNAs and DEGs **(C,D)**. The cutoff of DE-piRNAs was *p* < 0.01, FDR < 0.1, and absolute fold change >2. **(A,B)** The bar length represents the counts of target genes corresponding to the vertical terms. The bar color represents the *p*-adjust value of each term. BP, biological process; CC, cellular component; MF, molecular function; KEGG, Kyoto Encyclopedia of Genes and Genomes. **(C,D)** Pink nodes represent DE-piRNAs. Blue nodes represent DEGs. Nodes labeled have a node degree ≥5. The node size represents the node degree.

Similar to the analysis of DE-miRNAs, we performed GO and KEGG enrichment analysis for the target genes of DE-piRNAs. All of the significant enrichment analysis results we obtained are shown in Figures 4A,B. The genes targeted by upregulated piRNAs in HASN^A53T^ OX vs WT were enriched in post-embryonic development, regulation of locomotion and localization, cell surface receptor signaling pathway, response to stimulus, DNA conformation change and packaging, plasma membrane, RNA transport, axon regeneration, ATPase activity, and hydrolase activity (Figure 4A). The target genes of downregulated piRNAs for comparison of *tdp-1* KO vs WT were mainly enriched in proteasome assembly (target genes were D2045.2/H04D03.3, which were predicted to be targeted by 21ur-5985); mitophagy was also enriched with *fzo-1* gene (ortholog of human mitofusin 1, and mitofusin 2, involved in mitochondrial fusion), predicted to be targeted by 21ur-1080 (Figure 4B). Based on these results, the expression of piRNAs associated with the neuronal functions, such as regulation of locomotion and localization, axon regeneration, and hydrolase activity, might be induced by HASN^A53T^ OX and might cause a series of neuronal toxicities. *tdp-1* deletion caused piRNAs involved in proteasome assembly to be downregulated, which might promote the degradation of HASN^WT/A53T^ when *tdp-1* KO was crossed into HASN^WT/A53T^ OX strains. In addition, there were many differences between HASN^A53T^ OX and HASN^WT^ OX according to the enrichment analysis (Supplementary Figure S4). Post-embryonic development, chromosome organization, locomotion, DNA repair, endocytosis, autophagy, lysosome, synapse, axon regeneration, supramolecular complex, hydrolase activity, MAPK signaling pathway, mRNA surveillance pathway, mTOR signaling pathway, calcium signaling pathway, and longevity regulating pathway enrichments suggest that the piRNAs induced by HASN^A53T^ OX are distinct from HASN^WT^ OX.

To view the potential functional consequence of DE-piRNAs with a more relaxed criteria, we also performed GO and KEGG enrichment analysis for the target genes of DE-piRNAs with *p* value < 0.01 and absolute fold change > 2. More clusters were obtained compared with the previous stricter criteria of FDR < 0.1, *p* value < 0.01, and absolute fold change > 2 (Supplementary Figures S5–S7). For the comparison of HASN^A53T^ OX vs WT, more clusters associated with neurons were found from the upregulated DE-piRNAs: neurogenesis, neuron differentiation and development, regulation of nervous system development, and calcium signaling pathway. In addition, plasma membrane, endosome membrane, basement membrane, endocytosis, and autophagy were also enriched, which might be closely associated with the impairment of membranes induced by HASN^A53T^ OX (Supplementary Figure S5A), while supramolecular complex/polymer/fiber and hydrolase activity was found enriched for downregulated DE-piRNAs of comparison of HASN^A53T^ OX vs WT as well as regulation of nervous system development and regulation of axon guidance (Supplementary Figure S5B). Similar to the comparison of HASN^A53T^ OX vs WT, the target genes of DE-piRNAs of comparison of HASN^WT^ OX vs WT were mainly enriched in cytoskeleton (supramolecular complex), neuron projection cytoplasm, hydrolase activity, and ATPase activity (Supplementary Figures S6A,B). Regulation of locomotion, RNAi, myofibril, hydrolase activity, chromosome, homologous recombination, and protein dimerization activity were enriched for target genes of DE-piRNAs from the comparison of *tdp-1* KO vs WT (Supplementary Figures S7A,B). After the *tdp-1* KO was genetically crossed into the HASN^A53T^ OX strain, piRNAs involved in chromosome organization, morphogenesis, positive regulation of locomotion, muscle development, chromatin remodeling, supramolecular complex/polymer/fiber, synapse, bounding membrane of organelle, ErbB signaling pathway, MAPK signaling pathway, and calcium signaling pathway were dysregulated (Supplementary Figures S7C,D). Morphogenesis, supramolecular complex/polymer/fiber, hydrolase activity, helicase activity, ATPase activity, and cytoskeletal protein binding were enriched for DE-piRNAs of comparison of HASN^WT^ OX + *tdp-1* KO vs HASN^WT^ OX (Supplementary Figures S7E,F). Based on these results, piRNAs might contribute to the toxicities of HASN^A53T/WT^ OX on the nervous system, organelles membrane, and supramolecular complex. At the nuclear level, piRNAs might be involved in chromosome organization, chromatin remodeling, morphogenesis, locomotion, supramolecular complex/polymer/fiber, synapse, and calcium signaling pathway.

### Differentially Expressed MicroRNAs/Differentially Expressed PIWI-Interacting RNAs Might Be Associated With Several Neurodegenerative Diseases

To further evaluate the roles of DE-miRNAs/DE-piRNAs in neurodegenerative diseases, we explored the correlation between DE-miRNAs/DE-piRNAs target genes and human neurodegenerative diseases including AD, ALS, PD, MSA, and HD based on the gene–disease associations from the DisGeNET database. PD-associated gene *K07H8.2*, SLC41A1 ortholog in human, was predicted to be targeted by three DE-miRNAs: cel-miR-1018, cel-miR-230-3p, and cel-miR-797-5p. *tomm-40*, TOMM40 ortholog in human and related to AD, was predicted to be targeted by cel-miR-85-3p (Supplementary Table S7). Many DE-piRNAs from the comparison of HASN^A53T^ OX vs WT were found to be associated with PD including 21ur-10848, 21ur-11806, 21ur-295, 21ur-1412, 21ur-10487, 21ur-839, and 21ur-2781. Their predicted target genes were *gba-1* (GBA in human), *pgp-1* (ABCB1 in human), *T08G11.1* (VPS13A in human), *rab-39* (RAB39B in human), *tpa-1* (PRKCD in human), *taf-1* (TAF1 in human), *dop-3* (DRD2 in human), and *rme-8* (DNAJC13 in human). Among them, 21ur-1412 was predicted to target three genes related to neurodegenerative diseases PD or ALS, while 21ur-10487, 21ur-839, and 21ur-2781 were predicted to target two genes related to neurodegenerative diseases PD or AD (Supplementary Table S7).

### Differentially Expressed MicroRNAs/Differentially Expressed PIWI-Interacting RNAs Targeting Differentially Expressed Genes Related to Neuronal Function

We inspected the overlaps between the predicted target genes of DE-miRNAs (*p* value <0.05 and absolute fold change >2), DE-piRNAs (*p* value <0.01, absolute fold change >2, and FDR < 0.1), and the DEGs generated from the RNA-Seq of the same strains in our previous work with the cutoff of *p* value <0.05 and absolute fold change >2 (Shen et al., 2020). From the list of DE-miRNA/DE-piRNA target genes (Supplementary Tables S8, S9), the DE-miRNAs targeting the DEGs related to neuronal functions: autophagy, amino acid degradation, mitochondrion, and transcription activator were identified and listed in Table 1. Among these, cel-miR-1018, upregulated in the comparison of HASN^A53T^ OX vs WT, had its target DEG *T28F4.5* (linked to apoptotic signaling pathway and autophagy) downregulated. Cel-miR-355-5p, upregulated in the comparison of HASN^A53T^ OX vs WT and downregulated in the comparison of HASN^A53T^ OX + *tdp-1* KO vs HASN^A53T^ OX, had its target DEGs *C34F6.1* and *C05C10.3* down-/upregulated accordingly. Cel-miR-800-3p, upregulated in the comparison of HASN^A53T^ OX + *tdp-1* KO vs HASN^A53T^ OX, had its target DEG *camt-1* downregulated. PiRNA 21ur-1581, upregulated in the comparison of HASN^A53T^ OX vs WT, had its target DEG F54D10.3, which is related to dopaminergic neuron, downregulated. In addition, F54D10.3 was also confirmed as a target of piRNA by CLASH (crosslinking, ligation, and sequencing of hybrids) data (Shen et al., 2018) from piRTarbase. Based on these results, cel-miR-1018, cel-miR-355-5p, cel-miR-800-3p, 21ur-1581, and their targets *T28F4.5*, *C34F6.1*, *F54D10.3*, *C05C10.3*, and *camt-1* were suggested to be associated in the neurotoxicity of HASN^A53T^ OX.

**TABLE 1:** List of DE-miRNAs/DE-piRNAs targeting DEGs related to neuronal function.

Comparison	DE-miRNA/DE-piRNA (fold change)	DEG (fold change)	Description of DEG
HASN^A53T^ OX vs WT	cel-miR-1018 (2.32 ± 0.62)	*T28F4.5* (0.30 ± 0.08)	An ortholog of human DAP (death associated protein), regulation of catabolic process, apoptotic signaling pathway, regulation of autophagy.
	cel-miR-355-5p (2.95 ± 0.43)	*C34F6.1* (0.31 ± 0.14)	Predicted to have serine-type endopeptidase inhibitor activity.
	21ur-1581 (2.28 ± 0.10)	F54D10.3 (0.38 ± 0.05)	Enriched in PLM; carbon dioxide sensory neurons; dopaminergic neurons; germ line; and ventral nerve cord.
HASN^A53T^ OX + *tdp-1* KO vs HASN^A53T^ OX	cel-miR-355-5p (0.34 ± 0.03)	*C05C10.3* (2.40 ± 0.33)	An ortholog of human OXCT1 (3-oxoacid CoA-transferase 1), localizes to the mitochondrion.
	cel-miR-800-3p (2.41 ± 0.21)	*camt-1* (0.60 ± 0.10)	An ortholog of human CAMTA1 (calmodulin binding transcription activator 1) and CAMTA2. Proximal promoter DNA-binding transcription activator, RNA polymerase II-specific.

*Mean fold change of sRNA-seq was calculated by (mean of pseudo-count of group A)/(mean of pseudo-count of group B). The pseudo-count was calculated by edgeR. Fold change >1 denotes upregulated, and <1, downregulated. Data for DE-miRNA/DE-piRNA were obtained in the present study. Data for RNA-seq were obtained from Shen et al. (2020).DEG, differentially expressed gene.*

### Transcription Factor–MicroRNA–Gene Regulatory Pathways and Loops Were Screened From Networks of Differentially Expressed MicroRNAs, Differentially Expressed Genes, and Transcription Factors

**FIGURE 5:**
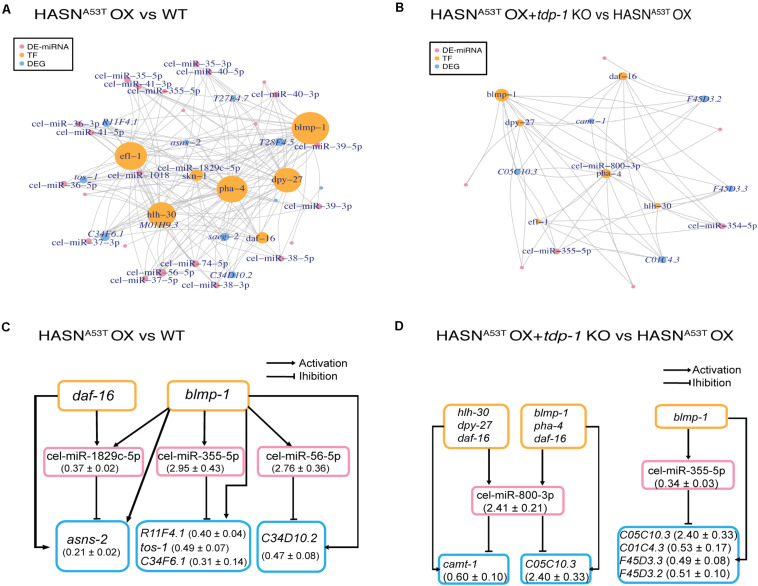
Networks of DE-miRNAs, DEGs (differentially expressed genes), and TFs (transcription factors). We obtained networks for only two comparisons: HASN^A53T^ OX vs WT and HASN^A53T^ OX + *tdp-1* KO vs HASN^A53T^ OX. DEGs were generated from the RNA-Seq of the same strains in our previous work with the cutoff of *p* value <0.05 and absolute fold change >2 (Shen et al., 2020). **(A,B)** Pink nodes represent DE-miRNAs. Orange nodes represent TFs. Blue nodes represent DEGs. The node size represents the node degree. Nodes labeled are the ones with a node degree >3. TFs were written as a vertical word in order to distinguish it from DEGs in these figures. **(C,D)** The pink rectangles indicate DE-miRNAs. The orange rectangles indicate TFs. The blue rectangles indicate DEGs. Fold change (mean ± SEM) of DE-miRNAs and DEGs are indicated in the parentheses.

To explore the TF–miRNA–gene regulatory pathways involved in the toxicity of HASN^A53T/WT^ OX with or without *tdp-1* in *C. elegans*, we constructed the networks of TFs, DE-miRNAs, and DEGs. We obtained networks for only two comparisons: HASN^A53T^ OX vs WT and HASN^A53T^ OX + *tdp-1* KO vs HASN^A53T^ OX (Figure 5). The nodes indicated in Figures 5A,B are those with a node degree >3. As shown in Figures 5A,B, *blmp-1* regulated the most DE-miRNAs and DEGs in both comparisons. *Efl-1*, *hlh-30*, *pha-4*, and *dpy-27* were also linked to many DE-miRNAs and DEGs for the comparison of HASN^A53T^ OX vs WT. Cel-miR-355-5p was the only miRNA identified for both comparisons with upregulation in HASN^A53T^ OX vs WT (2.95 ± 0.43, *p* = 9.71e−5) and downregulation in HASN^A53T^ OX + *tdp-1* KO vs HASN^A53T^ OX (0.34 ± 0.03, *p* = 4.64e−4). All the elements in the networks are listed in Supplementary Table S10.

Regulatory loops and pathways of TFs, DE-miRNAs, and DEGs were extracted from the networks in Figures 5A,B and displayed in Figures 5C,D and Supplementary Table S11. For comparison of HASN^A53T^ OX vs WT, six regulatory loops were built on two TFs, three DE-miRNAs, and five DEGs (Figure 5C). Based on the fold changes of DE-miRNAs and DEGs, *blmp-1* may have inhibited DEG *R11F4.1*, *tos-1*, *C34F6.1*, and *C34D10.2* expressions indirectly via activating cel-miR-355-5p and cel-miR-56-5p expressions in HASN^A53T^ OX. Furthermore, the TF-miRNA co-regulatory pair of *blmp-1* and cel-miR-355-5p was also involved in HASN^A53T^ OX + *tdp-1* KO vs HASN^A53T^ OX (Figure 5D). Accordingly, based on the fold changes, *C05C10.3* might be downregulated by this co-regulatory pair in HASN^A53T^ OX compared with HASN^A53T^ OX + *tdp-1* KO, while *hlh-30*, *dpy-27*, *daf-16*, *blmp-1*, and *pha-4* might inhibit *camt-1* expression via activating cel-miR-800-3p expression in HASN^A53T^ OX + *tdp-1* KO compared with HASN^A53T^ OX (Figure 5D). All of the TF–miRNA–gene regulatory pathways of these two comparisons with or without loops are listed row by row in Supplementary Table S11. The related lists for DE-miRNAs targeting DEGs, and TFs targeting DE-miRNAs are shown in Supplementary Tables S8 and S12 separately.

Due to the lack of a database for the interactions between TFs and piRNAs, we were not able to construct regulatory networks of TF–piRNA–gene. We constructed regulatory networks for DE-piRNAs and their target DEGs only (Figures 4C,D). As the cutoff we used for DE-piRNAs was *p* <0.01, FDR < 0.1, and absolute fold change >2, only comparisons HASN^A53T^ OX vs WT and *tdp-1* KO vs WT had results. Based on the ones exhibited in Figure 4C with node degree ≥5, there were eight DE-piRNAs – 21ur-6009, 21ur-3753, 21ur-6723, 21ur-1412, 21ur-6780, 21ur-2164, 21ur-2781, and 21ur-8060, targeting five DEGs or more. These piRNAs might play important roles in the toxicity of HASN^A53T^ OX. All of the DE-piRNAs targeting DEGs are listed in Supplementary Table S9.

### Novel MicroRNAs Were Predicted From sRNA-seq Data

miRDeep2 analysis identified a total of 293 novel miRNAs with significant randfold *p* values of which 34 novel miRNAs had a cutoff of miRDeep2 score ≥10 from all the sRNA-seq samples. The information for these novel miRNAs is listed in Supplementary Table S13.

### qRT-PCR Validations of Differentially Expressed MicroRNAs/Differentially Expressed PIWI-Interacting RNAs and Differentially Expressed Gene Candidates

To validate the expressions of DE-miRNAs/DE-piRNAs and DEGs, we performed qRT-PCR. The DE-miRNAs/DE-piRNAs and DEGs chose for qRT-PCR were mainly related to neuronal functions. The qRT-PCR results were compared with the sRNA-seq result of this study and the RNA-seq result in our previous study (Shen et al., 2020) and are shown in Table 2. The qRT-PCR results were consistent with our sRNA-seq/RNA-seq results, such as cel-miR-355-5p and its target DEGs. The expression of cel-miR-355-5p in comparison of HASN^A53T^ OX + *tdp-1* KO vs HASN^A53T^ OX was downregulated. Its corresponding target DEG *C05C10.3* was upregulated, while its expression in comparison of HASN^A53T^ OX vs WT was upregulated and its corresponding target DEG *C34F6.1* (serine-type endopeptidase inhibitor) was downregulated.

**TABLE 2:** qRT-PCR verification for sRNAs of sRNA-seq, and DEGs of RNA-seq.

Comparison	DE-miRNA/DE-piRNA/DEG	Fold change of sRNA-seq or RNA-seq	Fold change of qRT-PCR
HASN^A53T^ OX + *tdp-1* KO vs HASN^A53T^ OX	cel-miR-355-5p	0.34 ± 0.03	0.44 ± 0.03
HASN^WT^ OX vs WT	cel-miR-85-3p	0.50 ± 0.11	0.63 ± 0.32
HASN^A53T^ OX vs WT	cel-miR-355-5p	2.95 ± 0.43	1.47 ± 0.55
HASN^A53T^ OX vs WT	cel-miR-1018	2.32 ± 0.62	1.38 ± 0.19
HASN^A53T^ OX + *tdp-1* KO vs HASN^A53T^ OX	*camt-1*	0.6 ± 0.10	0.43 ± 0.21
HASN^A53T^ OX + *tdp-1* KO vs HASN^A53T^ OX	*C05C10.3*	2.40 ± 0.33	2.26 ± 0.92
HASN^A53T^ OX vs WT	*T28F4.5*	0.50 ± 0.15	0.30 ± 0.08
HASN^A53T^ OX vs WT	*C34F6.1*	0.31 ± 0.14	0.06 ± 0.02

*Mean fold change of RNA-seq was calculated by (mean of pseudo-count of group A)/(mean of pseudo-count of group B). The pseudo-count was calculated by edgeR. Fold change of qRT-PCR was calculated as follows: fold change_(a vs b)_ = 2^–[(CT of a – CT of Ref^^) – (CT of b – CT of Ref)]^.Ref, reference gene.All fold changes are shown as mean fold change of three samples ± SEM. Fold change >1 denotes upregulated, and <1, downregulated. Data for DE-miRNA/DE-piRNA and qRT-PCR were obtained in the present study. Data for RNA-seq were obtained from Shen et al. (2020).*

## Discussion

In this study, we observed that the miRNA/piRNA expression was broadly and abundantly altered by HASN^A53T^ OX, but not HASN^WT^ OX. TDP-1 likely supported the alterations by HASN^A53T^ OX since genetic crosses into *tdp-1* null mutants massively reduced the changes. Functional annotations for the target genes of DE-miRNAs/DE-piRNAs showed their effects on neuronal development and function. The relationship between the target genes and neurodegenerative diseases was also explored. The networks of TFs, DE-miRNAs, and DEGs, or DE-piRNAs and DEGs, predicted potential TF–miRNA–gene regulatory pathways and piRNA–gene regulatory pairs, which might play important roles in HASN^A53T^ OX toxicity.

The phenotypic differences between HASN^A53T^ and HASN^WT^ in humans are well established. The A53T mutation was first identified in a pedigree displaying early onset PD in a dominant mode of inheritance pattern (Polymeropoulos et al., 1997). Subsequent studies in model systems including our own show more severe phenotypes in animals with this specific mutation (Giasson et al., 2002; Gispert et al., 2003). HASN^A53T^ OX *C. elegans* animals had significant deficits including uncoordinated locomotion, impaired dopaminergic neurons, development and basal slowing response, and lengthened life span due to dietary restriction (Lakso et al., 2003; Vartiainen et al., 2006a; Shen et al., 2020). HASN^WT^ OX was similar to HASN^A53T^ OX in dopaminergic neuron loss and lengthened life span, but not in locomotion, development, and basal slowing response. There also existed differences in functional enrichments of their DEGs mainly in fatty acid and amino acid metabolism, immune response, endoplasmic reticulum unfolded protein response, posttranscriptional regulation of gene expression, enzyme activator activity, longevity regulating pathway, and the FoxO signaling pathway. In the present study, the target genes of DE-miRNAs/DE-piRNAs of HASN^A53T^ OX vs HASN^WT^ OX were mainly clustered in energy generation, amino acid metabolism, post-embryonic development, chromosome organization, locomotion, DNA repair, endocytosis, autophagy, lysosome, synapse, axon regeneration, supramolecular complex, hydrolase activity, MAPK signaling pathway, mRNA surveillance pathway, mTOR signaling pathway, calcium signaling pathway, and longevity regulating pathway. Hence, the differential effects of HASN^A53T^ OX and HASN^WT^ OX in *C. elegans* were also reflected by miRNA/piRNA expressions.

Although there are many different animal models constructed for PD research, those with α-synuclein overexpression in *C. elegans* have some distinctive advantages such as flexible genetic manipulation, a short life span, and a transparent body to facilitate the observation of specific phenotypes. Moreover, the α-synuclein overexpression *C. elegans* models can recapitulate some important neurochemical features of PD, such as the aggregation of α-synuclein and the degeneration of dopaminergic neurons (Gaeta et al., 2019). Many studies have furthermore shown promising translational outcomes in PD models. Ypt1p/Rab1 was screened in yeast as a suppressor of α-synuclein toxicity, which blocked vesicle trafficking between ER and Golgi. Its neuroprotective effect was then confirmed in animals including *Drosophila*, *C. elegans*, and neuronal cells from rat midbrain (Cooper et al., 2006). Proteins glucose-6-phosphate isomerase (GPI), VPS41 (involved in lysosomal biogenesis and Golgi-vacuole trafficking), and cathepsin D were identified in a screen as a protector against α-synuclein-mediated dopaminergic neuronal degeneration by RNAi in *C. elegans* and then verified in *Drosophila*, mice, and human neuroblastoma cells (Hamamichi et al., 2008; Qiao et al., 2008; Ruan et al., 2010; Knight et al., 2014). The chemical compound *N*-aryl benzimidazole (NAB) was obtained from a screen of α-synuclein overexpressing yeast cells and was also found to protect dopaminergic neurons from α-synuclein-mediated neurodegeneration in *C. elegans*, rat neurons, and human cells (Qiao et al., 2008). Together, these studies suggest that model organisms can be valuable tools in the translation for treatment of PD.

While our results indicate widespread changes in transcription involving hundreds of genes, it is possible that only a few genes or a single gene is important to facilitate the phenotypes observed. As an example, the PD-related gene *K07H8.2* was predicted to be targeted by cel-miR-1018 in the comparison of HASN^A53T^ OX vs WT and by cel-miR-230-3p and cel-miR-797-5p in the comparison of HASN^WT^ OX vs WT. *K07H8.2* was predicted to be targeted by different miRNAs in these two different types of HASN overexpression *C. elegans*, suggesting that it might be an important target in PD and related synucleinopathies. SLC41A1, a human ortholog of *K07H8.2*, is a Na^+^/Mg^2+^ exchanger responsible for the homeostasis of magnesium. Enhancement or loss of Mg^2+^-efflux induced by the dysfunction of SLC41A1 was found in PD patients and might trigger the following neurodegeneration (Kolisek et al., 2013; Lin et al., 2014; Wang et al., 2015). Our result is consistent with those of previous human studies.

An example of a single piRNA that could potentially have a significant impact is 21ur-10848, which was found to be upregulated (fold change = 4.15 ± 1.18, FDR = 0.014) in HASN^A53T^ OX compared with WT. Its predicted target gene is *gba-1*, an ortholog of human GBA (glucocerebrosidase, a lysosomal enzyme). GBA gene mutations are responsible for not only Gaucher’s disease (GD) and DLB but also PD (O’Regan et al., 2017; Gegg and Schapira, 2018; Riboldi and Di Fonzo, 2019). Its dysfunction causes metabolic imbalances of α-synuclein and other glucocerebrosidase substrates such as glucosylceramide.

We observed that *tdp-1* could influence dysregulation of miRNAs/piRNAs. TDP-43/TDP-1 has multiple functions in shaping the transcriptome including regulation of DNA transcription; splicing of pre-mRNA; translation, stabilization, and translocation of mRNA; and biogenesis of miRNA (Buratti and Baralle, 2012; Kawahara and Mieda-Sato, 2012). It also interacts with other proteins and regulates protein homeostasis via activating the TFs FOXOs (Buratti and Baralle, 2012; Zhang et al., 2014). The *tdp-1* product TDP-1 could support dysregulation of miRNAs through a number of direct or indirect mechanisms. A direct mechanism would be to control miRNA biogenesis via binding *C. elegans* orthologs of Drosha and/or Dicer (Kawahara and Mieda-Sato, 2012). Alternatively, it could bind miRNAs directly, although this binding is selective for specific miRNAs (Buratti et al., 2010; Paez-Colasante et al., 2020). Finally, it could act upstream during transcription of primary miRNAs as suggested by CHIP-seq studies, which show TDP-43 associated with miRNA transcription sites (Fan et al., 2014). Since TDP-43 globally regulates miRNA levels via these different mechanisms, and we observed many individual miRNA dysregulations, it is likely that one or more of these mechanisms take place. The regulatory mechanism of piRNAs by TDP-43 is more obscure. PiRNAs may be transcribed as primary piRNAs by noncanonical pol II actions or produced via transcription of transposons prior to amplification (Huang et al., 2017). The role of TDP-43 in any of these piRNA biogenesis pathways has not been studied deeply. A CHIP-seq study demonstrating TDP-43 binding at distinct piRNA loci suggests a pre-transcription control mechanism; however, that other roles similar to how miRNAs are regulated may also be possible (Fan et al., 2014), while *tdp-1* KO decreased the expression of α-synuclein according to our previous study (Shen et al., 2020). The decrease of dysregulated small RNAs might also be indirect due to the decreased expression of α-synuclein.

In this study, the target genes of TF–miRNA–gene regulatory loops, generated from both comparisons of HASN^A53T^ OX vs WT and HASN^A53T^ OX + *tdp-1* KO vs HASN^A53T^ OX, mainly functioned in amino acid metabolism (*C34F6.1*, *asns-2*, and *C05C10.3*), kinase activity (*R11F4.1* and *C01C4.3*), and transcription activation (*camt-1*). A TF–miRNA–gene forward and feedback regulation system might thus play a role in the toxicity of HASN^A53T^ OX. Whether piRNAs can form regulatory loops were not possible for us to determine in this study, as we were not able to directly link miRNAs and piRNAs in our network analysis. They may work cooperatively to regulate gene expression; however, there does not seem to be a great deal of overlap between miRNA and piRNA targets. Moreover, the role of transposons, the more well-established targets of piRNAs, in neurodegeneration is currently obscure. From the current study, it appears that miRNAs and piRNAs, though greatly dysregulated in our PD model, may independently regulate specific targets that together contribute to the observed neurodegeneration and behavioral phenotypes.

PiRNA works mainly in germline cells to protect the germline genome. As we used the whole body of the worm to perform the sRNA-seq, the dysregulated piRNAs in this study showed many target enrichment clusters related to post-embryonic development. However, some enrichments, which were related to neuronal toxicities, were also clustered for the targets of DE-piRNAs from the comparison of HASN^A53T^ OX vs WT, including regulation of nervous system development, axon regeneration, endocytosis, autophagy, hydrolase activity, and calcium signaling pathway. These results suggest the possibility of piRNA involvement in HASN^A53T^ OX neuronal toxicity. A more precise relationship between piRNAs, germline cells, and neurodegeneration would require additional studies.

This study profiled miRNAs and piRNAs for both HASN^A53T^ OX and HASN^WT^ OX based on the next-generation sequencing (NGS) techniques, which are sensitive and high-throughput (Behjati and Tarpey, 2013). We demonstrate abundant dysregulation, which is more predominant in HASN^A53T^ OX strains and also might depend upon *tdp-1* either directly through its own actions or indirectly through regulating α-synuclein or other neurodegeneration-related gene expressions. The dysregulated miRNAs/piRNAs and targets provide us with perturbed pathways and regulatory loops that together contribute to PD. Although we did not find evidence of cooperativity in miRNA and piRNA dysregulation, we did observe that their individual targets may independently contribute to PD and other neurodegeneration phenotypes. Additional layers of epigenetic complexity may also occur from other marks such as those from histones or in methylated DNA not studied here. These data thus provide insight into epigenetic mechanisms in PD that might be beneficial in development of potential therapies and diagnostic biomarkers for PD and related synucleinopathies.

## Data Availability Statement

The sRNA-seq raw data is available at SRA database under BioProject accession number, PRJNA649048. The datasets presented in this study can be found in online repositories. The names of the repository/repositories and accession number(s) can be found in the article/Supplementary Material.

## Author Contributions

LS designed and carried out the experiments, performed some of the sRNA-seq analysis, analyzed the data, and wrote the manuscript. CW performed most of the sRNA-seq analysis. LC performed some of the sRNA-seq analysis. GW conceived and supervised the project and edited the manuscript. All authors read and approved the final manuscript.

## Conflict of Interest

The authors declare that the research was conducted in the absence of any commercial or financial relationships that could be construed as a potential conflict of interest.

## Footnotes

1 https://www.ncbi.nlm.nih.gov/assembly/GCF_000002985.6

## Supplementary Material

Supplementary Figure 1 Hierarchical clustering of all miRNAs from strains with genotypes: WT, *tdp*-1 KO, and HASN^*WT*/*A53T*^ OX with or without *tdp*-1 gene. Hierarchical clustering was performed by complete linkage method with Euclidean distance. Logarithmic scale represents the range of expression values of miRNAs. The top cluster dendrogram indicates the similarity of different strains based on the expression values of all miRNAs of each strain. The left cluster dendrogram indicates the similarity of miRNAs based on the expression values of each miRNA of different strains. The right colored bars show the miRNAs expression values.

Supplementary Figure 2 **(A–M)** Volcano plots of miRNAs of different comparisons. The volcano plots were drawn by “plot” function and colored by “points” function of “graphics” package. The green nodes represent the miRNAs with *p* value <0.05 and absolute fold change >2. The orange nodes represent the miRNAs with absolute fold change >2 but *p* value ≥0.05. The red nodes represent the miRNAs with *p* value <0.05 but absolute fold change ≤2. The black nodes represent the miRNAs with *p* value ≥0.05 and absolute fold change ≤2.

Supplementary Figure 3 **(A–M)** Volcano plots of piRNAs from different comparisons. The volcano plots were drawn by “plot” function and colored by “points” function of “graphics” package. The green nodes represent the piRNAs with FDR < 0.1 and absolute fold change >2. The orange nodes represent the piRNAs with absolute fold change >2 but FDR ≥ 0.1. The red nodes represent the piRNAs with FDR < 0.1 but absolute fold change ≤2. The black nodes represent the piRNAs with FDR ≥ 0.1 and absolute fold change ≤2.

Supplementary Figure 4 **(A,B)** GO and KEGG enrichment analysis for the target genes of DE-piRNAs (*p* < 0.01, FDR < 0.1, and absolute fold change >2) from the comparison of HASN^*A53T*^ OX vs HASN^WT^ OX. The bar length represents the counts of target genes corresponding to the vertical terms. The bar color represents the *p*-adjust value of each term. BP, biological process; CC, cellular component; MF, molecular function; KEGG, Kyoto Encyclopedia of Genes and Genomes.

Supplementary Figure 5 **(A,B)** GO and KEGG enrichment analysis for the target genes of DE-piRNAs (*p* < 0.01 and absolute fold change >2) from the comparison of HASN^*A53T*^ OX vs WT. The bar length represents the counts of target genes corresponding to the vertical terms. The bar color represents the *p*-adjust value of each term. BP, biological process; CC, cellular component; MF, molecular function; KEGG, Kyoto Encyclopedia of Genes and Genomes.

Supplementary Figure 6 **(A,B)** GO and KEGG enrichment analysis for the target genes of DE-piRNAs (*p* < 0.01 and absolute fold change >2) from the comparison of HASN^WT^ OX vs WT. The bar length represents the counts of target genes corresponding to the vertical terms. The bar color represents the *p*-adjust value of each term. BP, biological process; CC, cellular component; MF, molecular function; KEGG, Kyoto Encyclopedia of Genes and Genomes.

Supplementary Figure 7 **(A–F)** GO and KEGG enrichment analysis for the target genes of DE-piRNAs (*p* < 0.01 and absolute fold change > 2) from the comparisons of *tdp*-1 KO vs WT, HASN^*A53T*^ OX + *tdp*-1 KO vs HASN^*A53T*^ OX, HASN^WT^ OX + *tdp-1* KO vs HASN^WT^ OX. The bar length represents the counts of target genes corresponding to the vertical terms. The bar color represents the *p*-adjust value of each term. BP, biological process; CC, cellular component; MF, molecular function; KEGG, Kyoto Encyclopedia of Genes and Genomes.

## Abbreviations

AD: Alzheimer’s disease
A β: β-amyloid
ALS: amyotrophic lateral sclerosis
BP: biological process
CC: cellular component
*C. elegans*: *Caenorhabditis elegans*
CLASH: crosslinking ligation and sequencing of hybrids
DEG(s): differentially expressed gene(s)
DLB: dementia with Lewy bodies
DE-miRNA: differentially expressed miRNA
DE-piRNA: differentially expressed piRNA
GO: Gene Ontology
NGS: next-generation sequencing
HASN^A53T^ OX: human α-synuclein A53T mutant overexpressing pan-neuronally
HASN^WT^ OX: human wild type α-synuclein overexpressing pan-neuronally
HASN^WT/A53T^ OX: human α-synuclein (WT or A53T) overexpressing pan-neuronally
HD: Huntington’s disease
KEGG: Kyoto Encyclopedia of Genes and Genomes
MF: molecular function
miRNA: microRNA
MSA: multiple system atrophy
PD: Parkinson’s disease
piRNA: PIWI-interacting RNA
sRNA-seq: small RNA sequencing
TarG: target gene
*tdp-1* KO: *tdp-1* knock out
TDP-43: Tar-DNA binding protein 43
TF(s): transcription factor(s)
WT: wild type.
