# Supplementary figures and images for: Dysregulation of MicroRNAs and PIWI-Interacting RNAs in a Caenorhabditis elegans Parkinson’s Disease Model Overexpressing Human α-Synuclein and Influence of tdp-1
Source: Front Neurosci. 2021 Mar 8;15:600462. doi: 10.3389/fnins.2021.600462 (PMC7982545; doi:10.3389/fnins.2021.600462)

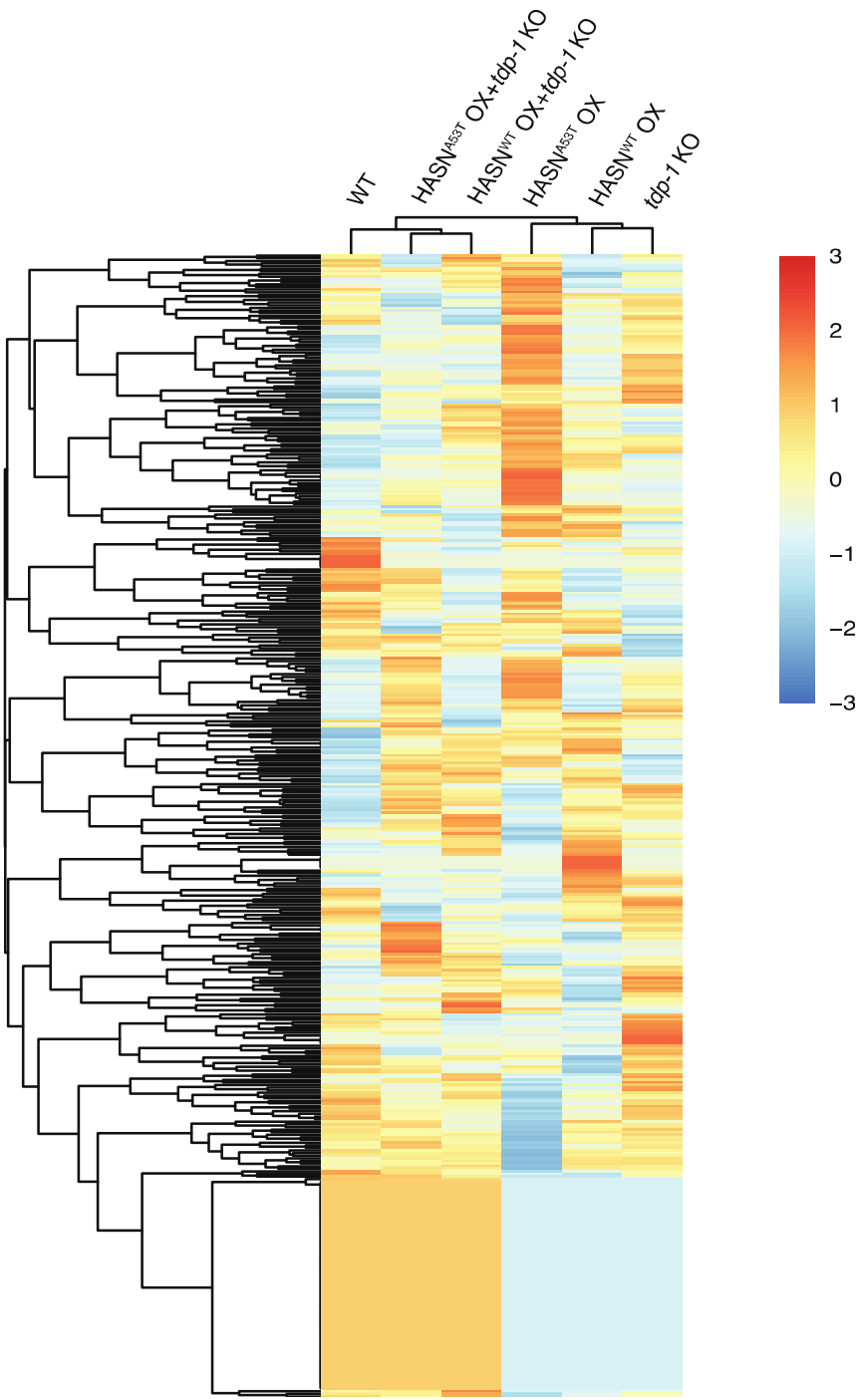

Supplement: Supplementary Figure 1 — Hierarchical clustering of all miRNAs from strains with genotypes: WT, tdp-1 KO, and HASNWT/A53T OX with or without tdp-1 gene. Hierarchical clustering was performed by complete linkage method with Euclidean distance. Logarithmic scale represents the range of expression values of miRNAs. The top cluster dendrogram indicates the similarity of different strains based on the expression values of all miRNAs of each strain. The left cluster dendrogram indicates the similarity of miRNAs based on the expression values of each miRNA of different strains. The right colored bars show the miRNAs expression values. [file Image_1.PDF]

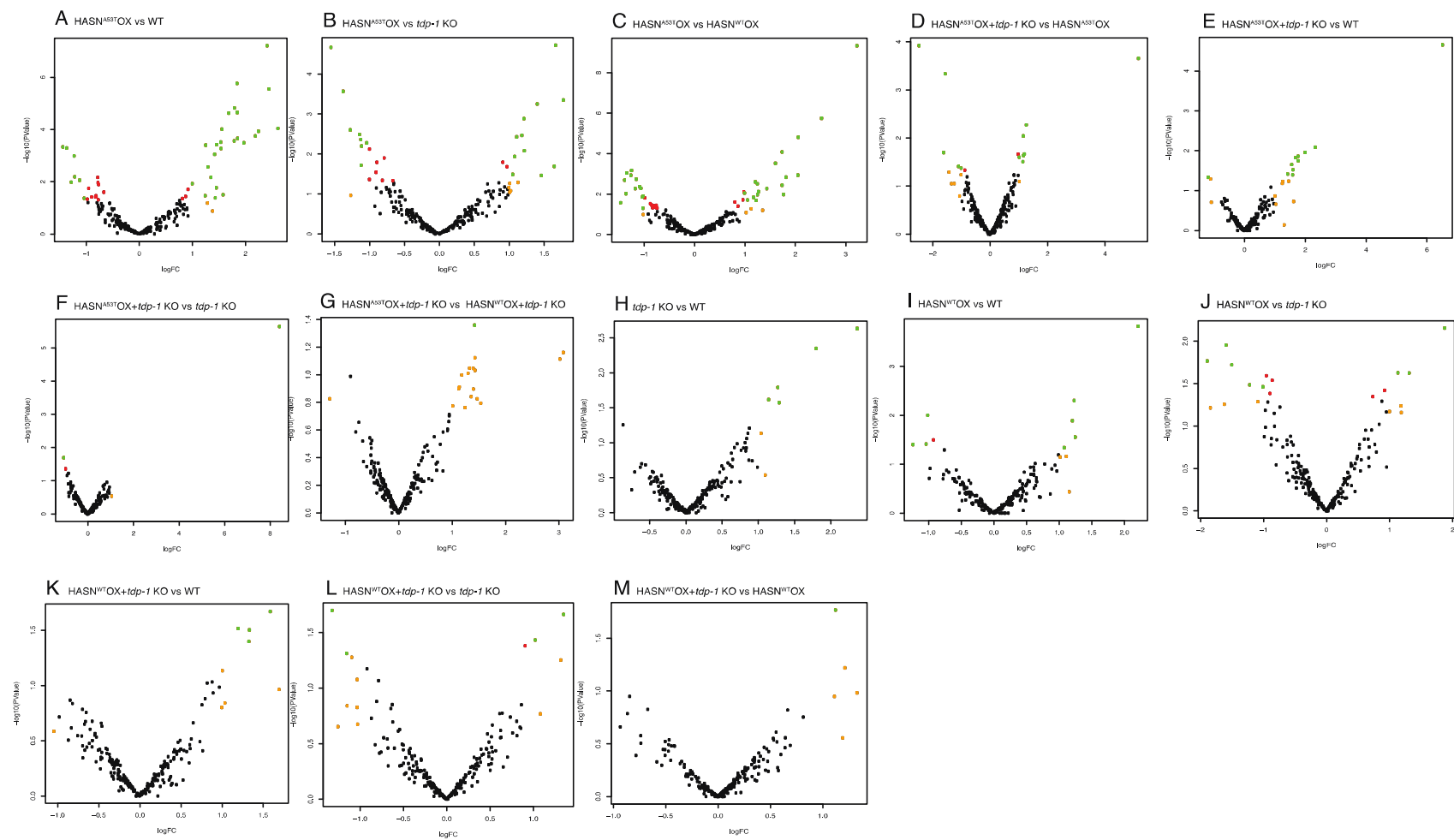

Supplement: Supplementary Figure 2 — (A–M) Volcano plots of miRNAs of different comparisons. The volcano plots were drawn by “plot” function and colored by “points” function of “graphics” package. The green nodes represent the miRNAs with p value <0.05 and absolute fold change >2. The orange nodes represent the miRNAs with absolute fold change >2 but p value ≥0.05. The red nodes represent the miRNAs with p value <0.05 but absolute fold change ≤2. The black nodes represent the miRNAs with p value ≥0.05 and absolute fold change ≤2. [file Image_2.PDF]

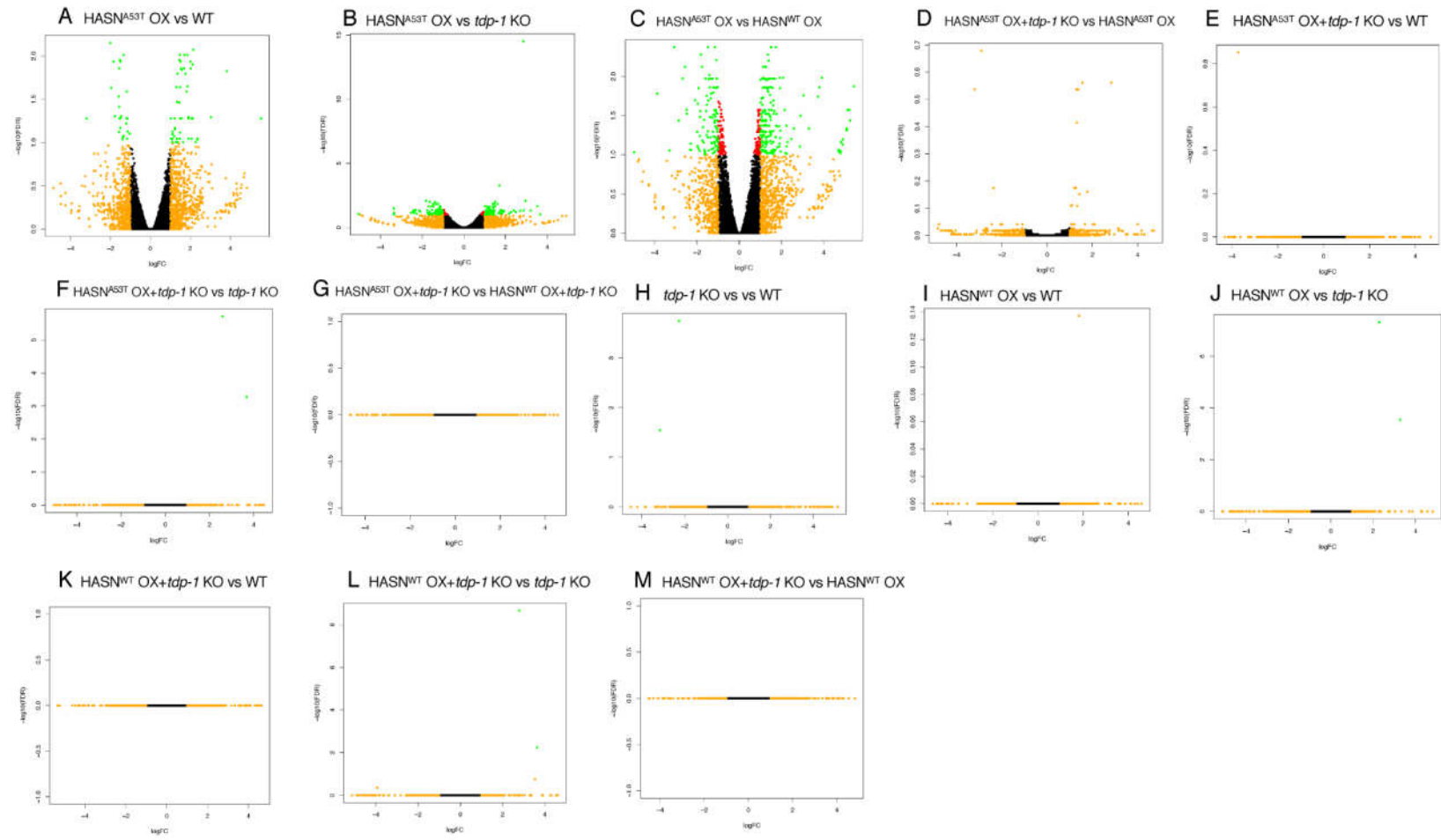

Supplement: Supplementary Figure 3 — (A–M) Volcano plots of piRNAs from different comparisons. The volcano plots were drawn by “plot” function and colored by “points” function of “graphics” package. The green nodes represent the piRNAs with FDR < 0.1 and absolute fold change >2. The orange nodes represent the piRNAs with absolute fold change >2 but FDR ≥ 0.1. The red nodes represent the piRNAs with FDR < 0.1 but absolute fold change ≤2. The black nodes represent the piRNAs with FDR ≥ 0.1 and absolute fold change ≤2. [file Image_3.PDF]
