# Supplementary material for: Dysregulation of MicroRNAs and PIWI-Interacting RNAs in a Caenorhabditis elegans Parkinson’s Disease Model Overexpressing Human α-Synuclein and Influence of tdp-1
Source: Front Neurosci. 2021 Mar 8;15:600462. doi: 10.3389/fnins.2021.600462 (PMC7982545; doi:10.3389/fnins.2021.600462)
Supplement: Supplementary Figure 4 — (A,B) GO and KEGG enrichment analysis for the target genes of DE-piRNAs (p < 0.01, FDR < 0.1, and absolute fold change >2) from the comparison of HASNA53T OX vs HASNWT OX. The bar length represents the counts of target genes corresponding to the vertical terms. The bar color represents the p-adjust value of each term. BP, biological process; CC, cellular component; MF, molecular function; KEGG, Kyoto Encyclopedia of Genes and Genomes. [file Image_4.PDF]

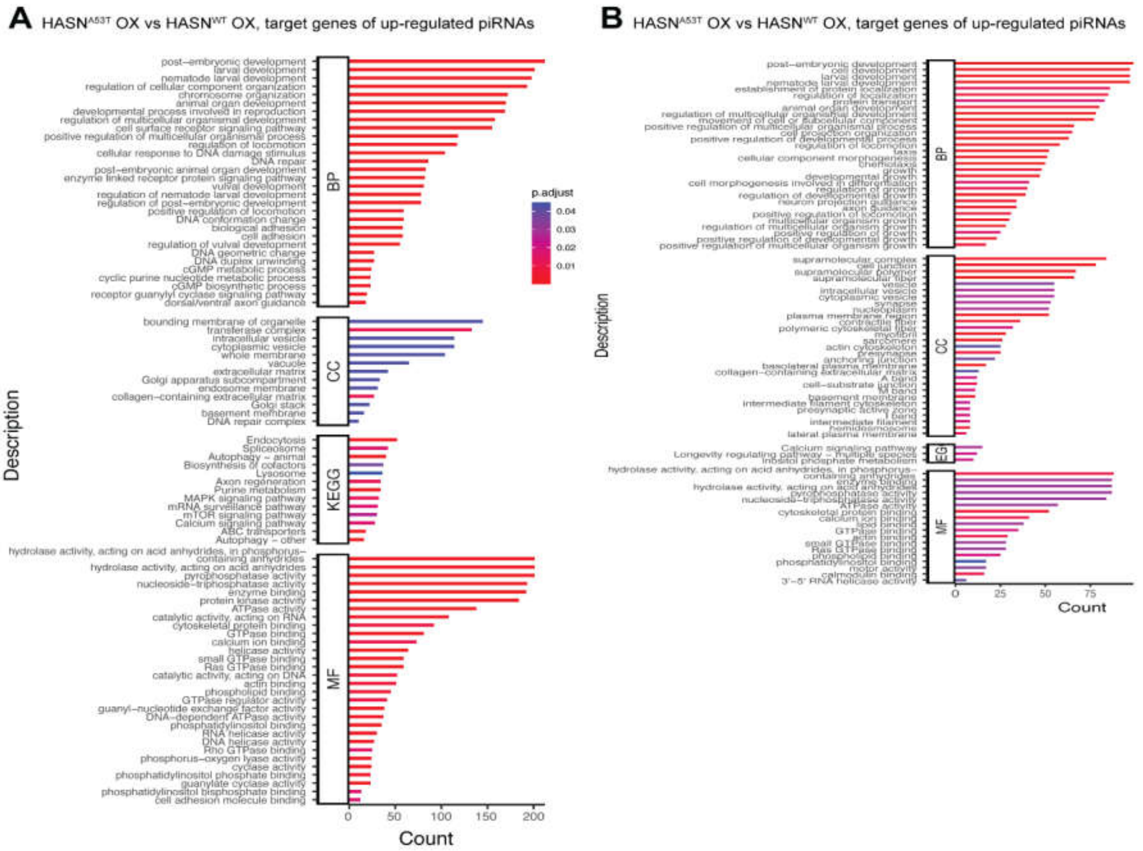
A
HASN^A53T OX vs HASN^WT OX, target genes of up-regulated piRNAs
Description
BP
CC
KEGG
MF
p.adjust
0.04
0.03
0.02
0.01
0
50
100
150
200
Count
B
HASN^A53T OX vs HASN^WT OX, target genes of up-regulated piRNAs
Description
BP
CC
EG
MF
p.adjust
0.04
0.03
0.02
0.01
0
25
50
75
Count
