# Supplementary material for: Dysregulation of MicroRNAs and PIWI-Interacting RNAs in a Caenorhabditis elegans Parkinson’s Disease Model Overexpressing Human α-Synuclein and Influence of tdp-1
Source: Front Neurosci. 2021 Mar 8;15:600462. doi: 10.3389/fnins.2021.600462 (PMC7982545; doi:10.3389/fnins.2021.600462)
Supplement: Supplementary Figure 5 — (A,B) GO and KEGG enrichment analysis for the target genes of DE-piRNAs (p < 0.01 and absolute fold change >2) from the comparison of HASNA53T OX vs WT. The bar length represents the counts of target genes corresponding to the vertical terms. The bar color represents the p-adjust value of each term. BP, biological process; CC, cellular component; MF, molecular function; KEGG, Kyoto Encyclopedia of Genes and Genomes. [file Image_5.PDF]

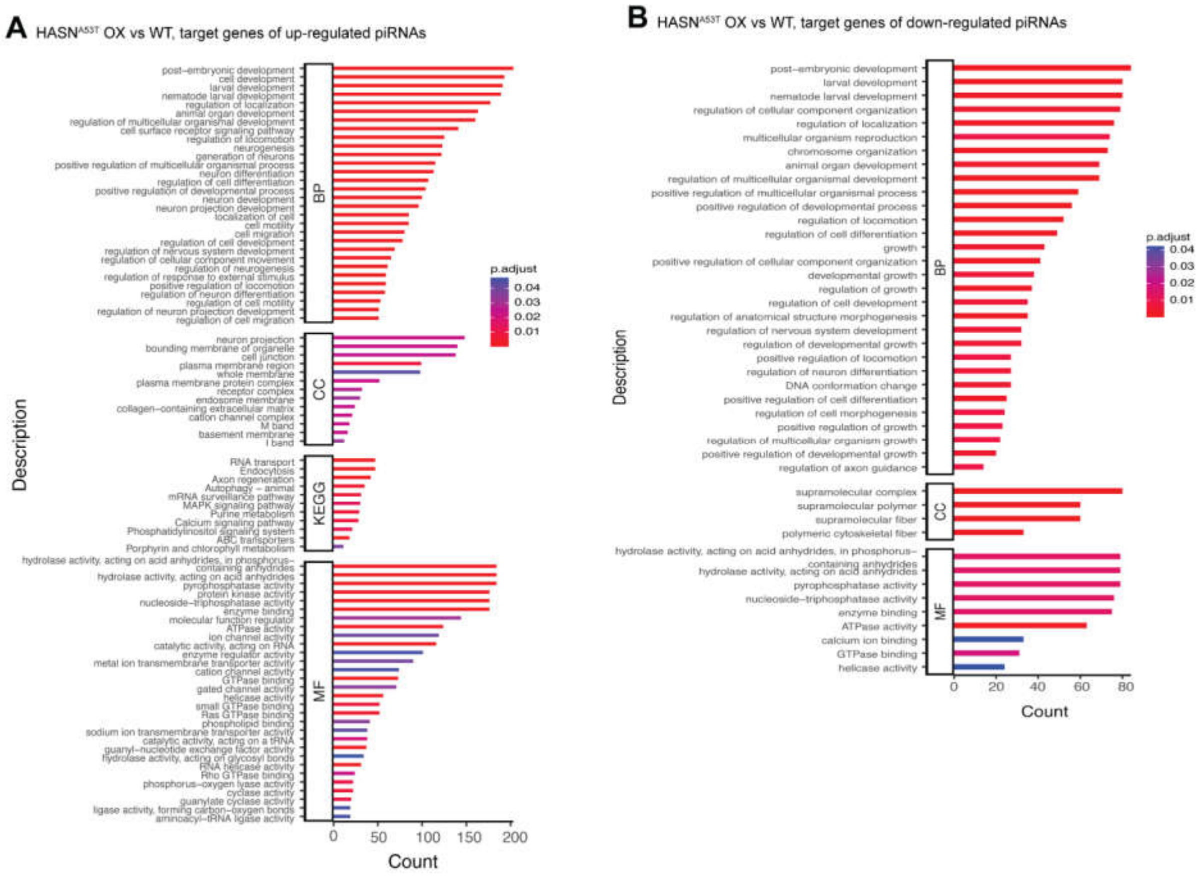
A HASNA53T OX vs WT, target genes of up-regulated piRNAs
Description
post–embryonic development
cell development
larval development
nematode larval development
regulation of localization
animal organ development
regulation of multicellular organismal development
cell surface receptor signaling pathway
regulation of locomotion
neurogenesis
generation of neurons
positive regulation of multicellular organismal process
neuron differentiation
regulation of cell differentiation
positive regulation of developmental process
neuron development
neuron projection development
localization of cell
cell motility
cell migration
regulation of cell development
regulation of nervous system development
regulation of cellular component movement
regulation of neurogenesis
regulation of response to external stimulus
positive regulation of locomotion
regulation of neuron differentiation
regulation of cell motility
regulation of neuron projection development
regulation of cell migration
BP
neuron projection
bounding membrane of organelle
cell junction
plasma membrane region
whole membrane
plasma membrane protein complex
receptor complex
endosome membrane
collagen–containing extracellular matrix
cation channel complex
M band
basement membrane
I band
CC
RNA transport
Endocytosis
Axon regeneration
Autophagy – animal
mRNA surveillance pathway
MAPK signaling pathway
Purine metabolism
Calcium signaling pathway
Phosphatidylinositol signaling system
ABC transporters
Porphyrin and chlorophyll metabolism
KEGG
hydrolase activity, acting on acid anhydrides, in phosphorus–containing anhydrides
hydrolase activity, acting on acid anhydrides
pyrophosphatase activity
protein kinase activity
nucleoside–triphosphatase activity
enzyme binding
molecular function regulator
ATPase activity
ion channel activity
catalytic activity, acting on RNA
enzyme regulator activity
metal ion transmembrane transporter activity
cation channel activity
GTPase binding
gated channel activity
helicase activity
small GTPase binding
Ras GTPase binding
phospholipid binding
sodium ion transmembrane transporter activity
catalytic activity, acting on a tRNA
guanyl–nucleotide exchange factor activity
hydrolase activity, acting on glycosyl bonds
RNA helicase activity
Rho GTPase binding
phosphorus–oxygen lyase activity
cyclase activity
guanylate cyclase activity
ligase activity, forming carbon–oxygen bonds
aminoacyl–tRNA ligase activity
MF
p.adjust
0.04
0.03
0.02
0.01
0
50
100
150
200
Count
B HASNA53T OX vs WT, target genes of down-regulated piRNAs
Description
post–embryonic development
larval development
nematode larval development
regulation of cellular component organization
regulation of localization
multicellular organism reproduction
chromosome organization
animal organ development
regulation of multicellular organismal development
positive regulation of multicellular organismal process
positive regulation of developmental process
regulation of locomotion
regulation of cell differentiation
growth
positive regulation of cellular component organization
developmental growth
regulation of growth
regulation of cell development
regulation of anatomical structure morphogenesis
regulation of nervous system development
regulation of developmental growth
positive regulation of locomotion
regulation of neuron differentiation
DNA conformation change
positive regulation of cell differentiation
regulation of cell morphogenesis
positive regulation of growth
regulation of multicellular organism growth
positive regulation of developmental growth
regulation of axon guidance
BP
supramolecular complex
supramolecular polymer
supramolecular fiber
polymeric cytoskeletal fiber
CC
hydrolase activity, acting on acid anhydrides, in phosphorus–containing anhydrides
hydrolase activity, acting on acid anhydrides
pyrophosphatase activity
nucleoside–triphosphatase activity
enzyme binding
ATPase activity
calcium ion binding
GTPase binding
helicase activity
MF
p.adjust
0.04
0.03
0.02
0.01
0
20
40
60
80
Count
