# Supplementary material for: Dysregulation of MicroRNAs and PIWI-Interacting RNAs in a Caenorhabditis elegans Parkinson’s Disease Model Overexpressing Human α-Synuclein and Influence of tdp-1
Source: Front Neurosci. 2021 Mar 8;15:600462. doi: 10.3389/fnins.2021.600462 (PMC7982545; doi:10.3389/fnins.2021.600462)

A HASN^{WT} OX vs WT, target genes of up-regulated piRNAs

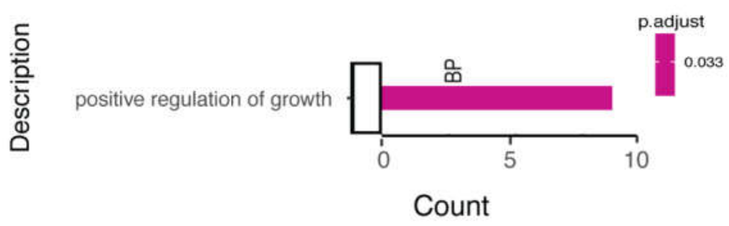

B HASN^{WT} OX vs WT, target genes of down-regulated piRNAs

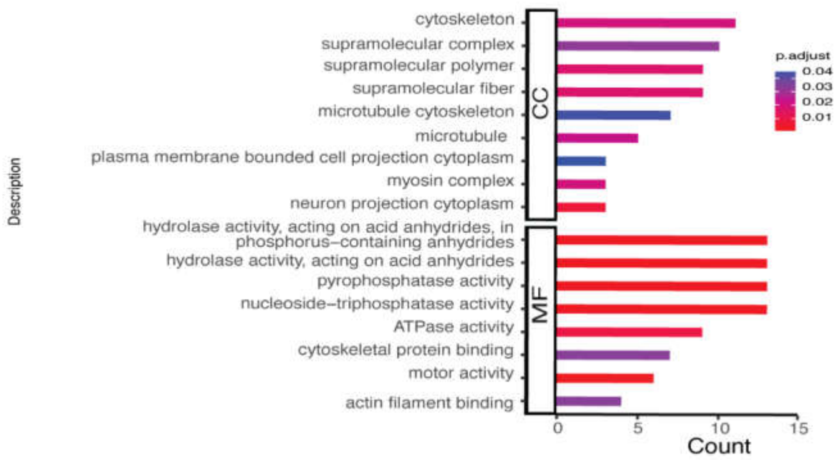

Supplement: Supplementary Figure 6 — (A,B) GO and KEGG enrichment analysis for the target genes of DE-piRNAs (p < 0.01 and absolute fold change >2) from the comparison of HASNWT OX vs WT. The bar length represents the counts of target genes corresponding to the vertical terms. The bar color represents the p-adjust value of each term. BP, biological process; CC, cellular component; MF, molecular function; KEGG, Kyoto Encyclopedia of Genes and Genomes. [file Image_6.PDF]
