# Supplementary material for: Dysregulation of MicroRNAs and PIWI-Interacting RNAs in a Caenorhabditis elegans Parkinson’s Disease Model Overexpressing Human α-Synuclein and Influence of tdp-1
Source: Front Neurosci. 2021 Mar 8;15:600462. doi: 10.3389/fnins.2021.600462 (PMC7982545; doi:10.3389/fnins.2021.600462)
Supplement: Supplementary Figure 7 — (A–F) GO and KEGG enrichment analysis for the target genes of DE-piRNAs (p < 0.01 and absolute fold change > 2) from the comparisons of tdp-1 KO vs WT, HASNA53T OX + tdp-1 KO vs HASNA53T OX, HASNWT OX + tdp-1 KO vs HASNWT OX. The bar length represents the counts of target genes corresponding to the vertical terms. The bar color represents the p-adjust value of each term. BP, biological process; CC, cellular component; MF, molecular function; KEGG, Kyoto Encyclopedia of Genes and Genomes. [file Image_7.PDF]

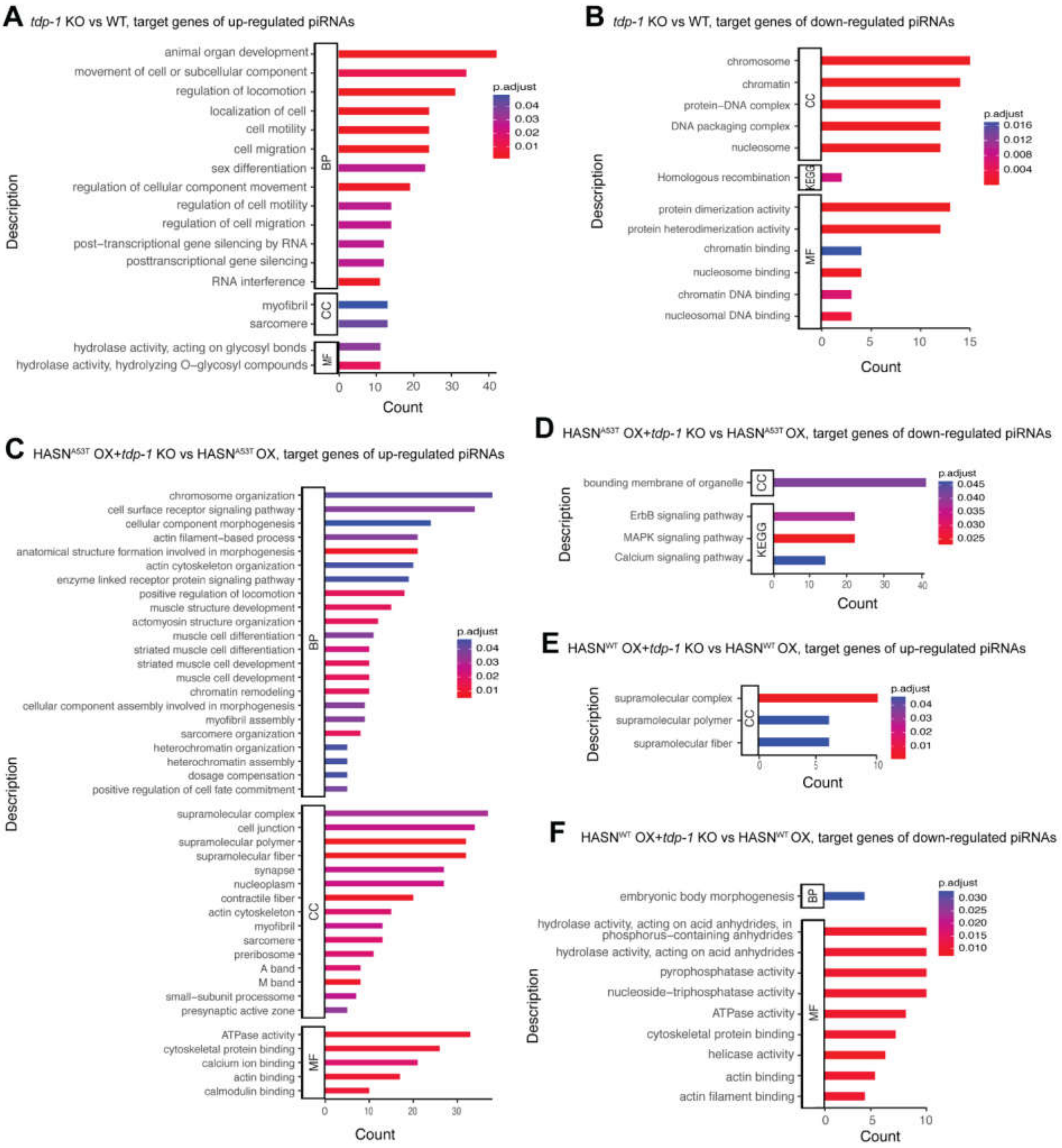
A tdp-1 KO vs WT, target genes of up-regulated piRNAs
animal organ development
movement of cell or subcellular component
regulation of locomotion
localization of cell
cell motility
cell migration
sex differentiation
regulation of cellular component movement
regulation of cell motility
regulation of cell migration
post−transcriptional gene silencing by RNA
posttranscriptional gene silencing
RNA interference
myofibril
sarcomere
hydrolase activity, acting on glycosyl bonds
hydrolase activity, hydrolyzing O−glycosyl compounds
BP
CC
MF
Description
Count
0
10
20
30
40
p.adjust
0.04
0.03
0.02
0.01
B tdp-1 KO vs WT, target genes of down-regulated piRNAs
chromosome
chromatin
protein−DNA complex
DNA packaging complex
nucleosome
Homologous recombination
protein dimerization activity
protein heterodimerization activity
chromatin binding
nucleosome binding
chromatin DNA binding
nucleosomal DNA binding
CC
KEGG
MF
Description
Count
0
5
10
15
p.adjust
0.016
0.012
0.008
0.004
C HASNA53T OX+tdp-1 KO vs HASNA53T OX, target genes of up-regulated piRNAs
chromosome organization
cell surface receptor signaling pathway
cellular component morphogenesis
actin filament−based process
anatomical structure formation involved in morphogenesis
actin cytoskeleton organization
enzyme linked receptor protein signaling pathway
positive regulation of locomotion
muscle structure development
actomyosin structure organization
muscle cell differentiation
striated muscle cell differentiation
striated muscle cell development
muscle cell development
chromatin remodeling
cellular component assembly involved in morphogenesis
myofibril assembly
sarcomere organization
heterochromatin organization
heterochromatin assembly
dosage compensation
positive regulation of cell fate commitment
supramolecular complex
cell junction
supramolecular polymer
supramolecular fiber
synapse
nucleoplasm
contractile fiber
actin cytoskeleton
myofibril
sarcomere
preribosome
A band
M band
small−subunit processome
presynaptic active zone
ATPase activity
cytoskeletal protein binding
calcium ion binding
actin binding
calmodulin binding
BP
CC
MF
Description
Count
0
10
20
30
p.adjust
0.04
0.03
0.02
0.01
D HASNA53T OX+tdp-1 KO vs HASNA53T OX, target genes of down-regulated piRNAs
bounding membrane of organelle
ErbB signaling pathway
MAPK signaling pathway
Calcium signaling pathway
CC
KEGG
Description
Count
0
10
20
30
40
p.adjust
0.045
0.040
0.035
0.030
0.025
E HASNWT OX+tdp-1 KO vs HASNWT OX, target genes of up-regulated piRNAs
supramolecular complex
supramolecular polymer
supramolecular fiber
CC
Description
Count
0
5
10
p.adjust
0.04
0.03
0.02
0.01
F HASNWT OX+tdp-1 KO vs HASNWT OX, target genes of down-regulated piRNAs
embryonic body morphogenesis
hydrolase activity, acting on acid anhydrides, in phosphorus−containing anhydrides
hydrolase activity, acting on acid anhydrides
pyrophosphatase activity
nucleoside−triphosphatase activity
ATPase activity
cytoskeletal protein binding
helicase activity
actin binding
actin filament binding
BP
MF
Description
Count
0
5
10
p.adjust
0.030
0.025
0.020
0.015
0.010
